# Medical Education

**Published:** 1932

**Authors:** C. E. S. Flemming

**Affiliations:** Bradford-on-Avon


### MEDICAL EDUCATION.

BY

C. E. S. Flemming, M.R.C.S., L.R.C.P.,

*Bradford-on-Avon.*

A few years ago Dame Janet Campbell's *Report on Maternal Mortality* caused much concern among the profession and the public, and an official inquiry showed that there were various causes for the unsatisfactory state of midwifery practice there set out, one of the most important being defective education. One wonders what would be the result of a similar inquiry with regard to medicine generally; it would probably show, there also, a serious inadequacy with one of its causes the system of education of medical men, with this difference: that while in midwifery it is starvation and neglect, in medicine it is over-anxiety and engorgement. In medicine the errors are, generally speaking, not so obvious, the disasters not so dramatic, as in midwifery. The fault is not primarily with the practitioner; he has to take what is given him. The onus lies heavily on those responsible for his training and qualification. We must, however, remember that with the rapid advance in medicine much more is expected and required, and our system of education has not been able properly to accommodate itself to the changes that have taken place.

The newly-qualified man of to-day is no doubt more efficient than was his predecessor of forty years ago, but his ability to make use of his knowledge is no better, if as good. Probably, with his greater knowledge at the moment of starting practice, he has less need to use his powers of observation and deduction, and if these fail him as his lately-acquired knowledge fades, will his wisdom grow ?

Unfortunately, we cannot shut our eyes to the fact that excellent as are the medical practitioners of to-day, there are yet too many whose ignorance and incapacity are only cloaked by dogmatism and craft, and others whose want of exercise of their reasoning powers has caused them to fail in professional efficiency.

If, as is the case in the great majority, proficiency has been attained, has there not been much waste of money that could ill be afforded, and, worse still, waste of time, waste of too much of the best space of life, time spent in useless compulsory lectures, in acquiring unwanted knowledge, in examinations and the preparation for them ? Briefly, time and money have not been spent to the best advantage, and opportunities have been lost.

One of the serious faults of medical education is the putting an exaggerated value on mere knowledge as apart from reason, the attempt to learn too many facts, to keep pace with new knowledge while failing to give up or to defer the teaching of what is obsolete or not wanted at the moment.

A further important defect is the division of study into watertight compartments, the want of association of one part with another, so that when the student passes from one compartment to another he is apt to shut out of his mind what he learnt in the first. For instance, anatomy and physiology are taught for two years with practically no reference to clinical facts ; by the time a medical student gets into the ward, how much of his examination anatomy does he remember or need ? Still more does this apply when he gets into practice. Knowledge is, of course, essential, but dry facts without an appreciation of their import can never be interesting. The block system of teaching is the result of a similar system of examinations, and if one is to be altered, so must the other be.

Unfortunately, examinations have become a fetish. It is now a question of training primarily for examination, and incidentally for practice, whereas it should be training primarily for practice and incidentally for examination. The result is that studies are designed with the examiner in view and not the patient. Remarkable evidence of this is the publication of numberless books written especially for examination purposes. Why is this necessary ?

By encouraging an accumulation of facts rather than methods of observation and deduction, examinations defeat the aim of teaching to think. What actually is the value of examinations as conducted to-day ? Do they show us anything of the practical competence of the examinee ? Is it possible in a few questions to find out what we really want to find out ?

Why, it is asked, change our system if the British is the best in the world ? We must change because, though the best, it is far from being perfect, because medicine is constantly changing and advancing, and education to be effective must keep pace with it, and because there is much to be learned from the systems of other countries, even if they are not in every way better than ours.

What is the remedy ? We must first know what we want ? The public want practical men, men whom they can consult on every subject concerning their health, who will tell them what they ought to do and when it is necessary for them to seek further advice. The public do not want essay writers only; not, for example, the highly-qualified medical man who could not set a simple greenstick fracture in the forearm of a child, though he became later, in a small area of the body, a specialist of some repute.

The national medical service of the nation wants competent practitioners, general practitioners first. Sir George Newman has told us lately how important is the practitioner in the medical service of the country. The general practitioner can, if necessary, become a specialist; the man who has been trained only to be a specialist can never become a general practitioner, who is not only a potential specialist but is also, if he has been properly trained, a scientific researcher, a practitioner of preventive medicine, with unique opportunities of studying the earliest stages and the life history of disease.

The qualification of a general practitioner is fundamental to the making of a specialist, that is if he is to treat human beings and not merely isolated bits of their bodies.

There is no foundation whatever for the idea that any one medical man cannot know enough to give his patient the best value of medicine and when to call in the expert. Incidentally, are we not apt to forget that the doctor has in his books a long array of consultants? And, after all, the majority of illnesses met with in practice do not require the assistance of a specialist.

If we have decided that what we have to train and qualify is a general medical practitioner, how are we to start? We must consider who are to be the students. If it is useless to waste first-class education on third-class persons, do not abolish the first-class education but the third-class person. There is no doubt that under the British system some unfit men are admitted as students, men of unsuitable character or of insufficient intelligence. In America there is a regulated system of selection and many are rejected, but it is doubtful whether this practice would suit us in Great Britain. The selection of candidates for naval cadetships, as is done by a special Board, is a method that might be better. The present system of the survival of the fittest is no doubt wasteful of educating machinery, and some form of selection after the first year of study might be practicable.

Sir George Newman, fourteen years ago, wrote: "We require a man of high character, able and willing to maintain the true dignity of a great profession, and to live up to the high ethical traditions of medicine; of good general education, of interest, activity and some *savoir-faire;* a man of learning and knowledge in his vocation, with technical skill and medical experience; but, above all, a practical man, with ideas and application, with a forward looking mind, able to participate in the growth and development of medical science, trained in the scientific method and inspired by the scientific spirit; in other words, a man of accurate observation, of ability in experimentation, and of sound judgment and interpretation."

Having found the man we want, how are we to improve his education? Not by adding to the length of the curriculum, which has already reached the practical, economical, physical and mental limit, and is out of proportion to the result or need.

We must give up the attempt to do the impossible in learning endless facts, remembering, however, at the same time that practical medicine is the application of many sciences. There must be constant revision as to what is to be added to and what is to be excised from undergraduate studies ; but trouble comes when decision has to be made as to what shall be omitted. It will be necessary for some independent body to determine this ; for while all concerned agree that economy is necessary, each in turn says : "Not in my department."

We want a higher standard for entrance, a command of the English language, so that expression may be given clearly to our thoughts by speech or by writing. There must be some knowledge of the fundamentals : physics, chemistry (inorganic and organic), and biology, not only because of their relation to every branch of medicine, but also in order that the training they bring of scientific methods, exact observation and logical deduction may be thoroughly inculcated and colour the whole of the student's professional work both before and after qualifying. In his professional studies, in particular sections of more strictly medical work, attention will naturally be drawn to the importance of those subjects. This continuity and association of subjects must pertain throughout the whole curriculum. There must be nothing useless ; each subject must be considered in its proper place and proportion. First will come a study of the body, its form and function—anatomy and physiology—but not in too much detail. In surgery, medicine and midwifery the special details of these subjects should be learned as their need and application become obvious ; thus will this knowledge be appreciated and retained, and for this reason the sooner some sort of clinical work begins the better, even in the first year. When work begins in the wards it is apt to be forgotten how necessary it is to have a knowledge of the body in health, and to realize what wide variations of form and function are consonant with health.

There is little doubt that too much time and attention is given to surgery, not its general principles, which are essential, but its specialized details. Much time is wasted in the theatre and the lecture-room acquiring knowledge and skill of a kind that is only required by the specialist, and too little, much too little, time is given to medicine, both in the wards and the out-patient departments. Here clinicians are too apt to miss the constant opportunities of teaching the study of man as part of the study of disease, and illustrating by clinical example the reasoned deductions of books and lectures.

There is too great a temptation to demonstrate and lecture on the less common things, and to slur over the things of daily occurrence, which are, in fact, not only more essential to practice, but often much more difficult to explain because of the habit of taking them for granted. Throughout the curriculum it is necessary to remember that it is a general practitioner that is being made, not a specialist—that specialist study can or should only be made after qualification.

The question of lectures is important ; there are far too many that are compulsory. The having to attend so many, the signing up, are an evil influence that pervades all study and should undoubtedly be done away with. They are the heritage of an old order that should be dealt with by an iconoclast. Good lectures, good demonstrations, clinical or other, need no compulsion for attendance ; if they are not good they are a serious waste of time. Compulsory lectures give the bad lecturer too good a chance. It goes without saying that skill in surgery or eminence in medicine does not necessarily mean skill in teaching.

Good lecturers are none too common; they are born, not made, and the birth-rate is not a high one.

Are examinations, as now held, necessary? A uniform qualifying final examination can hardly be abolished, and other examinations for diplomas of special distinction or special branches of medicine should be taken after the qualification. There is not space here to discuss the reason, but it is probably accepted generally that the one-portal system is the ideal; unfortunately vested and local interests stand in the way of its adoption. The examination itself might quite well be held in the various teaching centres. It should be, so far as possible, practical. Also it should be required that all the subjects necessary for qualification be passed at the one time. If the system of examination is satisfactory there need be no hardship. If a man can only pass in three subjects by taking one at a time, it means that he cannot carry in his mind at the same time the other two, so that if the examination is to be a test of his capacity he is, strictly speaking, only fit to practise in the subject last passed; either this is so or the method of examination is wrong.

It is, no doubt, generally recognized that examinations are unsatisfactory, and it should not pass human wit to find a better means of testing capacity. An expert obstetrician lately said that in half an hour's practical examination he could test the capacity of a midwife better than with pages of papers.

Not enough, if any, consideration is given to the knowledge of the teacher of the capacity, in every respect, of the candidate he has taught. Such a practice might allow of favouritism, but that would be checked by the final examination by an outside body; even if not, it is no more open to objection than the element of chance which enters so largely into the present system. It is the intermediate examinations that distract the student, waste his time, misdirect his energies and interfere with the ordered continuity that there should be in his studies. Is there any reason why they should not be held by the school itself ?

It might well be the rule that men could only proceed from one period of education to another after passing a local examination, and this should be conducted by the men who, by their practical experience, know what the student should know of the subjects in which he is being examined. For instance, before a student becomes eligible for work in the ward as dresser or clinical clerk he should have passed an examination in anatomy and physiology held by physicians, surgeons, and obstetricians, as they must know better than the professors of anatomy and physiology what of these subjects he will require in the practice of surgery, medicine and obstetrics. The education and examination of the men who are to be entrusted with the lives and health of the nation have been determined to too great an extent by those who are not in close contact with general practice and cognizant of its requirements.

Education, of course, never finishes, certainly not with the final examination. But at what period of his education does a man become efficient enough to practise alone ? It is the attempt to pass out men fully qualified for practice that causes the curriculum to be so crowded. Most men who have been some years in practice will say that he is certainly not fit when he has just passed ; many men look back and wonder how they had the temerity to practise without first doing at least one resident job. The right to practise alone should not be granted until at least a year has been spent in residence at some hospital, where the novitiate may learn the technical application of his recently-acquired knowledge. Against this it is argued that there are not enough jobs for all, but with the numerous municipal hospitals now coming into being there must be many more opportunities, and, as a matter of fact, at the present time many institutions are finding considerable difficulty in getting residents.

No system of education can be considered complete without provision for postgraduate study, and many schemes are being formulated and put into practice to make it generally available; but none can be more effective than the provision of hospital beds, where the general practitioner can not only treat many of his patients, but also come into contact with colleagues and consultants, and so help to keep himself conversant with current methods and ideas.

Many councils are already discussing the means whereby medical education may be improved, but it does seem desirable that some disinterested committee should inquire into the whole question and report to the General Medical Council, which must be the ultimate authority. Probably the British Medical Association, in touch as it is with all parts of the profession, with its experience and influence, would be the most appropriate and useful body to set up a small and authoritative committee.

REFERENCES.

Sir George Newman, *Notes on Medical Education in England*. London, 1918, H.M.S.O.

Professor Berry, *Methods and Objectives of Medical Education*. 1929. (See page 247 of present issue).

C. M. Wilson, "The Student in Irons," *Brit. Med. Jour.*, 1932, i. 485.

H. S. Souttar, "The Education of the Medical Student," *Brit. Med. Jour.*, 1932, ii. 427.

*St. Mary's Hospital Gazette*, April–June, 1932.

